# Practical Application of a New Cuffless Blood Pressure Measurement Method

**DOI:** 10.3390/pathophysiology30040042

**Published:** 2023-12-01

**Authors:** Nana Gogiberidze, Aleksandr Suvorov, Elizaveta Sultygova, Zhanna Sagirova, Natalia Kuznetsova, Daria Gognieva, Petr Chomakhidze, Victor Frolov, Aleksandra Bykova, Dinara Mesitskaya, Alena Novikova, Danila Kondakov, Alexey Volovchenko, Stefano Omboni, Philippe Kopylov

**Affiliations:** 1 Department of Cardiology, Functional and Ultrasound Diagnostics of N.V. Sklifosovsky Institute for Clinical Medicine, I.M. Sechenov First Moscow State Medical University (Sechenov University), 119991 Moscow, Russia; zhanna.s.n@mail.ru (Z.S.); dashkagog@mail.ru (D.G.); petr7747@mail.ru (P.C.); aabykowa@yandex.ru (A.B.); aranid980@gmail.com (D.M.); alena.ru89@inbox.ru (A.N.); den14712@gmail.com (D.K.); dr.volovchenko@mail.ru (A.V.); stefano.omboni@iitelemed.org (S.O.); fjk@inbox.ru (P.K.); 2 World-Class Research Center “Digital Biodesign and Personalized Healthcare”, I.M. Sechenov First Moscow State Medical University (Sechenov University), 119991 Moscow, Russia; suvorov_a_yu_1@staff.sechenov.ru (A.S.); sultygovaliza@gmail.com (E.S.); tusia.13@bk.ru (N.K.); 3 Medical Center for Premorbid and Emergency Conditions, P.V. Mandryka Central Military Clinical Hospital, 121002 Moscow, Russia; v.frolov-med@mail.ru; 4 Italian Institute of Telemedicine, Via Colombera 29, 21048 Solbiate Arno, Varese, Italy

**Keywords:** blood pressure, cuffless, blood pressure measurement, portable ECG monitor, CardioQVARK device, telemedicine

## Abstract

It would be useful to develop a reliable method for the cuffless measurement of blood pressure (BP), as such a method could be made available anytime and anywhere for the effective screening and monitoring of arterial hypertension. The purpose of this study is to evaluate blood pressure measurements through a CardioQVARK device in clinical practice in different patient groups. Methods: This study involved 167 patients aged 31 to 88 years (mean 64.2 ± 7.8 years) with normal blood pressure, high blood pressure, and compensated high blood pressure. During each session, three routine blood pressure measurements with intervals of 30 s were taken using a sphygmomanometer with an appropriate cuff size, and the mean value was selected for comparison. The measurements were carried out by two observers trained at the same time with a reference sphygmomanometer using a Y-shaped connector. In the minute following the last cuff-based measurements, an electrocardiogram (ECG) with an I-lead and a photoplethysmocardiogram were recorded simultaneously for 3 min with the CardioQVARK device. We compared the systolic and diastolic BP obtained from a cuff-based mercury sphygmomanometer and smartphone-case-based BP device: the CardioQVARK monitor. A statistical analysis plan was developed using the IEEE Standard for Wearable Cuffless Blood Pressure Devices. Bland–Altman plots were used to estimate the precision of cuffless measurements. Results: The mean difference between the values defined by CardioQVARK and the cuff-based sphygmomanometer for systolic blood pressure (SBP) was 0.31 ± 3.61, while that for diastolic blood pressure (DBP) was 0.44 ± 3.76. The mean absolute difference (MAD) for SBP was 3.44 ± 2.5 mm Hg, and that for DBP was 3.21 ± 2.82 mm Hg. In the subgroups, the smallest error (less than 3 mm Hg) was observed in the prehypertension group, with a slightly larger error (up to 4 mm Hg) found among patients with a normal blood pressure and stage 1 hypertension. The largest error was found in the stage 2 hypertension group (4–5.5 mm Hg). The largest error was 4.2 mm Hg in the high blood pressure group. We, therefore, did not record an error in excess of 7 mmHg, the upper boundary considered acceptable in the IEEE recommendations. We also did not reach a mean error of 5 mmHg, the upper boundary considered acceptable according to the very recent ESH recommendations. At the same time, in all groups of patients, the systolic blood pressure was determined with an error of less than 5 mm Hg in more than 80% of patients. While this study shows that the CardioQVARK device meets the standards of IEEE, the Bland–Altman analysis indicates that the cuffless measurement of diastolic blood pressure has significant bias. The difference was very small and unlikely to be of clinical relevance for the individual patient, but it may well have epidemiological relevance on a population level. Therefore, the CardioQVARK device, while being worthwhile for monitoring patients over time, may not be suitable for screening purposes. Cuffless blood pressure measurement devices are emerging as a convenient and tolerable alternative to cuff-based devices. However, there are several limitations to cuffless blood pressure measurement devices that should be considered. For instance, this study showed a high proportion of measurements with a measurement error of <5 mmHg, while detecting a small, although statistically significant, bias in the measurement of diastolic blood pressure. This suggests that this device may not be suitable for screening purposes. However, its value for monitoring BP over time is confirmed. Furthermore, and most importantly, the easy measurement method and the device portability (integrated in a smartphone) may increase the self-awareness of hypertensive patients and, potentially, lead to an improved adherence to their treatment. Conclusion: The cuffless blood pressure technology developed in this study was tested in accordance with the IEEE protocol and showed great precision in patient groups with different blood pressure ranges. This approach, therefore, has the potential to be applied in clinical practice.

## 1. Introduction

Hypertension, or high blood pressure, is one of the major risk factors for stroke, other cardiovascular diseases (CVD), chronic kidney disease, and dementia. Blood pressure refers to the pressure exerted on the walls of blood vessels by blood flowing through these blood vessels. A high blood pressure is the strongest modifiable risk factor for cardiovascular disease worldwide [1,2,3,4,5,6]. Monitoring blood pressure (BP) is critical to identify and adequately treat this important cardiovascular risk factor [7]. A reliable assessment of blood pressure (BP) allows one to detect any deviations from normal values that may indicate a disease and can also be used to evaluate the effectiveness of antihypertensive therapy. The gold standard for evaluation of systolic and diastolic blood pressure is an invasive assessment of the central arterial blood pressure. Due to the invasive approach, the risk of complications is significant [8]. Blood pressure devices currently in use are predominantly based on the oscillometric method. This measurement method provides intermittent readings rather than continuous monitoring and may deliver inaccurate measurements for various reasons such as different cuff sizes [9,10,11,12,13,14]. Non-invasive wireless monitoring systems are an appealing development that could offer wider applications in different settings and facilitate telemedicine monitoring of blood pressure.

Photoplethysmography (PPG) is a method of optics based on changes in blood volume during the heart cycle in peripheral arterioles [15]. The existing pulse transit time (PTT) method uses an ECG sensor for the heart and a photoplethysmography sensor when measuring other peripheral parts. Photoplethysmography can observe changes in blood flow by optically detecting light reflected or transmitted from tissues and blood. Based on the R-peak measured in the electrocardiogram, either the time difference between the start points of the pulse wave of the photoplethysmography signal or the time difference between the points when the PPG signal is used; these measurements have maximum values of PTTb and PTTt, respectively [16,17]. The pulse transit time (PTT) is known to be an indicator of the BP level and may be the key to cuffless BP measurement [16,17,18], depending on its determination from ECG and photoplethysmography data [19,20,21,22]. Various models have been used for BP assessments based on the photoplethysmography method [23]. One study used a CardioQVARK device, a smartphone case that offers simultaneous recording of the electrocardiogram and provides a continuous recording of the photoplethysmography image of the pulse wave. All received data were registered on the server, based on which an algorithm used to measure blood pressure was built [24]. The IEEE (Institute of Electrical and Electronics Engineers) standards map has previously been used for practical measurements with the CardioQVARK device [25,26,27]. The aim of this paper is to validate a method for non-invasive blood pressure measurement based on the IEEE Standard for Wearable Cuffless Blood Pressure Devices [28].

## 2. Materials and Methods

This is a prospective observational study that was conducted at the I.M. Sechenov First Moscow State Medical University (Sechenov University, Moscow, Russia), Clinical Hospital №1 in Moscow, Russia, between December 2020 and November 2021. This study was conducted according to the guidelines of the Declaration of Helsinki and approved by the Local Ethics Committee of I.M. Sechenov First Moscow State Medical University (Sechenov University), protocol code NO. 14–19. All participants gave written informed consent.

### 2.1. Study Patients

The sample size was determined according to the IEEE Standard for Wearable Cuffless Blood Pressure Measuring Devices [28]. The inclusion criteria were age >18 years and written informed consent of the patient. The exclusion criterion was poor quality of the ECG or pulse wave. This study included patients with a normal blood pressure and patients with hypertension or a compensated high blood pressure who achieved the target blood pressure level during the treatment of arterial hypertension with an increase of 2–3 degrees.

### 2.2. Blood Pressure Measurement and Data Acquisition

In the first phase of the study, the observers were trained. Two observers were trained in the accurate measurement of blood pressure and familiarized themselves with the data collection procedure and the operation of the device [28]. In the main study phase, the blood pressure used in the analysis was measured by a trained observer following the British Hypertension Society (BHS) protocols [26,27]. Three measurements were taken in the seated position, and the average value was used as the BP input to determine the subject’s BP classification. The patient sat quietly for 15 min before the measurement. The cuff was placed on the left upper arm, 2 cm above the elbow. During each session, we took 3 cuff blood pressure measurements at 30 s intervals using a sphygmomanometer. We used a properly sized cuff. The cuff was inflated until the pressure that it exerted on the underlying arm was high enough to stop blood flow underneath the cuff, such that no blood flow sounds could be heard. As the cuff pressure was reduced, the pressure transmitted from the cuff to the walls of the underlying arteries was reduced until blood flow resumed and the sound of blood flow could again be heard. These sounds can vary in intensity and usually stop at the point of the lowest pressure within the arteries before the next pulse arrives. The mean value of 3 measurements was selected for further analysis. The measurements were carried out by two observers trained at the same time with a reference sphygmomanometer (using a Y-shaped connector). Systolic blood pressure (SBP) and diastolic blood pressure (DBP) measurements with the mercury sphygmomanometer were determined, respectively, using the Phase 1 and Phase 5 Korotkoff sounds. If the measurements of the two observers were no more than 4 mm Hg from each other, the mean values of the two observers were used as a reference.

**Figure 1:**
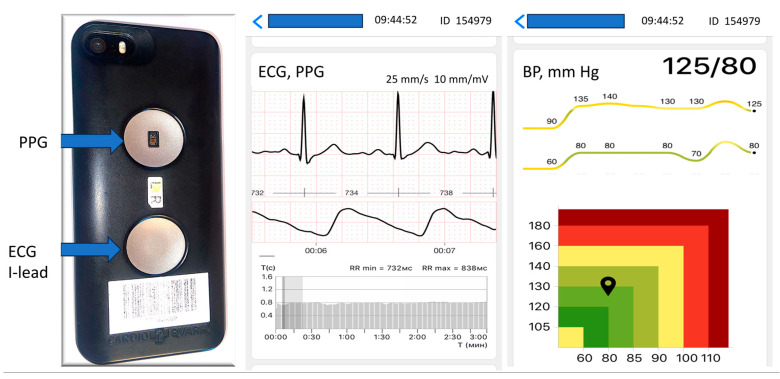
Characteristics of the CardioQVARK device. The left side presents the electrode for I-lead ECG registration, and the right side shows the monitor for the photoplethysmography PPG. The device is presented together with an example of a recording.

Within one minute of the cuff-based measurement series, an I-lead ECG with simultaneous photoplethysmocardiogram was recorded over 3 min with a CardioQVARK device (Figure 1). ECG signals were recorded from the fingers using one ECG lead. The sensors provided a continuous recording of the photoplethysmography image of the pulse wave, synchronized with the electrocardiogram cycles. The algorithm we used in this study was based on simultaneous evaluation of the electrocardiogram and pulse transit time parameters, which were recorded with a smartphone case. The device and application were combined into one unit and registered with the Federal Service for Surveillance in Healthcare № RZN 2019/8124 on 15 February 2019. Detailed characteristics and the working algorithm of the CardioQVARK device have been previously reported [24].

### 2.3. Statistical Analysis

We compared the systolic and diastolic blood pressure obtained from the cuff-based mercury sphygmomanometer and smartphone-case-based BP device (CardioQVARK monitor).

Descriptive statistics for the numerical data included the mean (M), standard deviation (SD), median, minimum, maximum, and 2.5, 25, 75, and 97.5 percentiles. Normality was assessed using a Shapiro–Wilk test. For categorical data, the proportions and absolute values were determined.

A blood pressure assessment with the Korotkoff method was used as the reference method.

The statistical analysis plan was obtained from the Institute of Electrical and Electronics Engineers Standard for Wearable Cuffless Blood Pressure Measuring Devices [28].

Bland–Altman plots were used to estimate the precision of cuffless measurements. The mean difference (MD), mean absolute difference (MAD), and mean absolute percentage difference (MAPD) (CI) were calculated with a 95% confidence interval:

$$MD=\frac{\sum_{i=1}^{n}new_{i}-ref_{i}}{n}$$

$$MAD=\frac{\left(\sum_{i=1}^{n}\left|new_{i}-ref_{i}\right|\right)}{n}$$

$$MAPD=\frac{100\times \left(\sum_{i=1}^{n}\left|new_{i}-ref_{i}\right|/ref_{i}\right)}{n}.$$

Cumulative percentages and cumulative distribution functions were estimated for the MAD. Cumulative percentages were calculated for MADs of ≤5, ≤10, and ≤15 mmHg. Histograms were drawn for a visual assessment of the MD.

Statistical analysis was conducted using SPSS v. 23 and R v.4.0. The Bland–Altman plot was used to test agreement between the two measurement methods, where the cuff-based mercury sphygmomanometer method was the reference method.

## 3. Results

This study included 167 patients, 64 women and 102 men, from 31 to 88 years of age (mean 64.2 ± 7.8 years). In total, 61.1% were males (Table 1). In addition, gender, date of birth, medical history, and medications were recorded on the case report form (Table 2). Patients with different BP levels were included (Table 1).

The mean systolic blood pressure (SBP) among our patients was 130.5 ± 23.0 mm Hg (range 88–191 mm Hg), and the mean diastolic blood pressure (DBP) was 81.5 ± 13.8 mm Hg (range 54–122 mm Hg) after applying the cuff-based mercury sphygmomanometer (Table 2). The mean SBP was 128.3 ± 17.9 mm Hg (range 87–188 mm Hg), and the mean DBP was 79.2 ± 11.2 mm Hg (range 56–121 mm Hg) when measured using the CardioQVARK monitor.

The aim of this paper was to validate a method for non-invasive blood pressure measurements based on the IEEE Standard for Wearable Cuffless Blood Pressure Devices. The IEEE Standard for Wearable Cuffless Blood Pressure Devices foresees a two-phase validation process. The first phase (Table 3) requires a minimum of 20 subjects, and the second phase requires an additional 25 subjects (a total of at least 45 subjects is required). In each stage, the measurement error must be evaluated separately for the entire group and in each age subgroup to determine systolic and diastolic blood pressure. The error is estimated based on the mean difference (MD), mean absolute difference (MAD), and mean absolute difference in percentage (MAPD) and then ranked using the ANSI/AAMI SP10 and BHS scales [29,30]. In the case of sufficient rankings, the method can be recommended for use. Vascular compliance is a key determinant of wave propagation in the vascular system. Hence, pulse wave velocity measurements are used as a method for detecting vessel stiffness using the VaSera VS-1500N [31] (Table 2). The VaSera VS-1500N device non-invasively measures blood pressure in four limbs with simultaneous recording of ECG, PCG, and pulse waves in the carotid, femoral arteries, and arteries of the four limbs. Thus, a sphygmometer makes it possible to study the distensibility of the arteries and the degree of blood flow disturbance in the vessels of the patient’s lower extremities.

An analysis of the device accuracy is presented in Table 4 according to the IEEE-SA Standards.

Basic descriptive statistics for errors are provided in the Appendix A in Table A1, Table A2, Table A3, Table A4 and Table A5.

**Figure 2:**
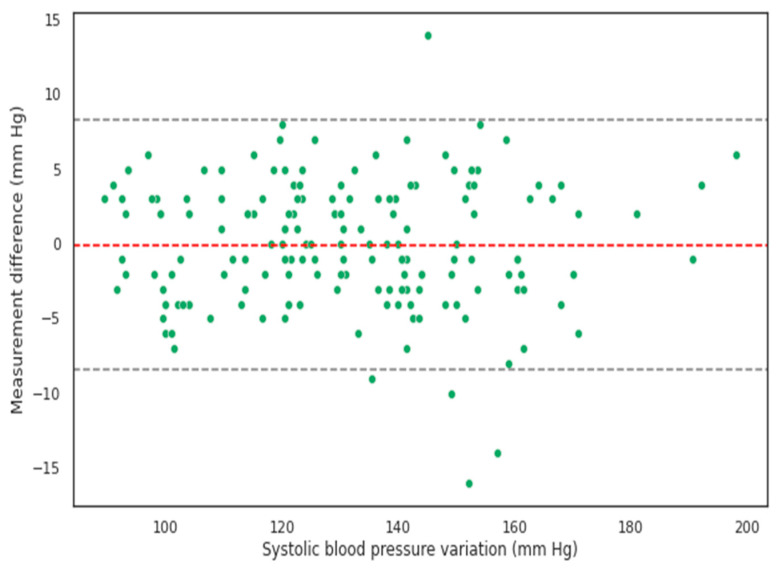
Bland–Altman graph of systolic blood pressure values derived from cuffless measurements versus cuff-based mercury sphygmomanometer measurements. Here, the raw mean difference is −0.05 [−0.7, 0.6] mm Hg. The error values are distributed homogeneously along the x-axis, with the proportion of values exceeding 1.96*SD being extremely small.

**Figure 3:**
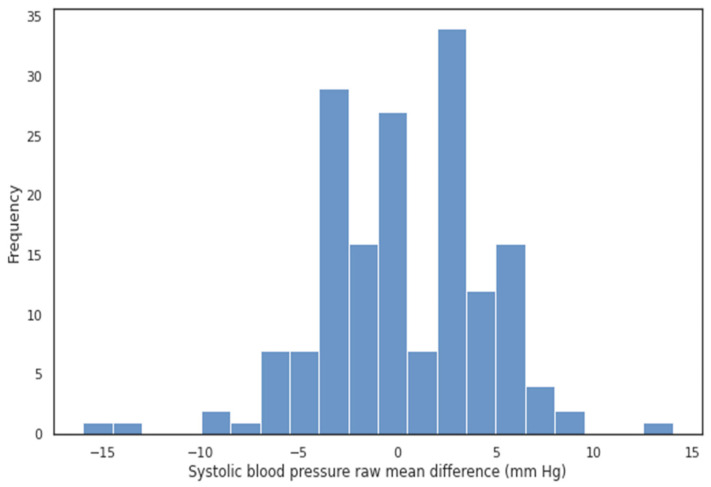
Histogram of raw MD for systolic blood pressure. The density plots suggest a roughly normal distribution, with the majority of values lying inside the [−5; 5] mm Hg interval. The cumulative percentage for MAD ≤ 5 mm Hg encompassed 85.6% of values.

**Figure 4:**
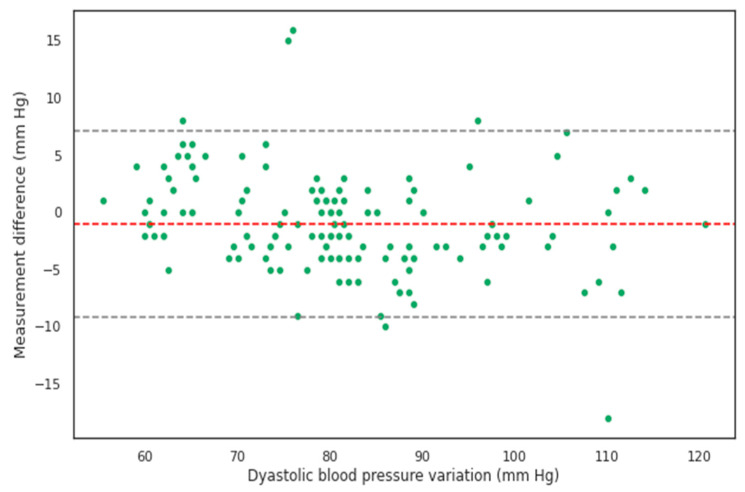
Bland–Altman plot of diastolic blood pressure values derived from cuffless measurements versus cuff-based mercury sphygmomanometer measurements. The raw mean difference was −1.02 [−1.65; −0.38].

**Figure 5:**
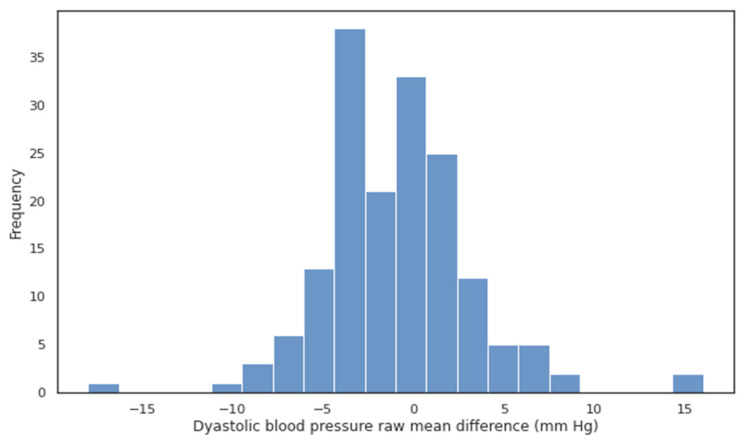
Histogram of raw MD for diastolic blood pressure (DBP). There is a slight underestimation of the DBP; the raw MD was −1.02 [−1.65; −0.38]. The cumulative percentage for MAD ≤ 5 mm Hg constituted 83.8% of values.

The Bland–Altman analysis showed that the SBP and DBP values calculated using the BP device without a cuff matched the values measured using a mercury sphygmomanometer with a cuff (Figure 2, Figure 3, Figure 4 and Figure 5).

## 4. Discussion

Developing cuffless methods for the remote monitoring of blood pressure is a valuable undertaking since such technologies have the potential to improve blood pressure control. We previously tested a new algorithm for BP determination using ECG and PPG parameters recorded with a smartphone case against oscillometric BP measurements taken in a large sample of hypertensive patients [24]. In the present study, we compared measurements using a CardioQVARK device with measurements using a cuff-based mercury sphygmomanometer according to the standards of The Institute of Electrical and Electronics Engineers for Wearable and Cuffless Blood Pressure Measuring Devices [14,18,20]. This study included patients of different age groups and with different blood pressure levels (Table 1) and overcame some of the limitations of our previous study [24].

The mean difference between the measurements using CardioQVARK and those using the cuff-based mercury sphygmomanometer for systolic blood pressure was −0.05 ± 4.25 mm Hg, while the difference for diastolic blood pressure was −1.02 ± 4.15 mm Hg. The mean absolute difference (MAD) for systolic blood pressure was 3.44 ± 2.5 mm Hg, while that for diastolic blood pressure was 3.21 ± 2.82 mm Hg.

In the subgroups, the smallest error (less than 3 mm Hg) was observed in the prehypertension group, with a slightly larger error (up to 4 mm Hg) found among normal blood pressure and stage 1 hypertension patients. The largest error was observed in the stage 2 hypertension group (4–5.5 mm Hg). The largest error was 4.2 mm Hg in the high blood pressure group. We, therefore, did not record an error in excess of 7 mmHg, the upper boundary considered acceptable for the IEEE recommendations. We also did not reach a mean error of 5 mmHg, the upper boundary considered acceptable according to the recent ESH recommendations [32]. At the same time, in all groups of patients, the systolic pressure was correctly determined with an error of less than 5 mm Hg in more than 80% of patients.

Overall, while this study shows that the CardioQVARK device meets the standards of the IEEE, the Bland–Altman analysis indicates that the cuffless measurement of diastolic blood pressure retains a significant bias. The difference was very small and unlikely to be of clinical relevance for the individual patient, but this may well have epidemiological relevance on a population level. Therefore, the CardioQVARK device, while being worthwhile for monitoring patients over time, may not be suitable for screening purposes.

An algorithm proven to correctly determine blood pressure was integrated into a mobile phone case [24]. The great advantage of such a method is that the patient requires no additional devices, only a smart phone. Cuffless blood pressure measurement devices are emerging as a convenient and tolerable alternative to cuff-based devices [33]. However, there are several limitations to cuffless blood pressure measurement devices that should be considered. For instance, this study showed a high proportion of measurements with a measurement error of <5 mmHg, while detecting a small, although statistically significant, bias in the measurement of diastolic blood pressure. This suggests that this device may not be suitable for screening purposes. However, its value for monitoring BP over time is confirmed [34,35]. Furthermore, and most importantly, the easy measurement method and the device portability (integrated in a smartphone) may increase the self-awareness of hypertensive patients and, potentially, lead to an improved adherence to their treatment.

### Limitations

There are some limitations to this study. First, our study used the IEEE Standard for Wearable Cuffless Blood Pressure Devices to validate the non-invasive blood pressure measurement method instead of the ESC, the corresponding clinical society with recent recommendations (2023) and more stringent criteria. Second, the sample size was determined according to the IEEE Standard for Wearable Cuffless Blood Pressure Devices (this number is lower than the sample size currently recommended in the most recent ESH recommendations) [32]. Thirdly, the use of the proposed device is limited in epidemiological studies to evaluate cut-off values for screening, for which small BP differences have been shown to potentially have a significant public health impact. The device may be adequate for blood pressure monitoring over time, however. Finally, the blood pressure measurements were consecutive and not simultaneous. However, the comparatively brief interruption in time likely did not lead to a substantial loss of information.

## 5. Conclusions

In this study, the cuffless blood pressure measuring technology we developed was tested according to the IEEE protocol and showed a high accuracy in groups of patients with different blood pressure ranges. This approach, therefore, has the potential to be applied in clinical practice.

## Figures and Tables

**Table 1:** Blood pressure in cohort patients as measured by oscillometric measurements.

Current SBP (mm Hg)	Mean SBP in Group (mm Hg)	Number of Patients	Age	Male (%)
All	130.5 ± 23.0	167	65.3 ± 11.3	61.1
≥160	168.9 ± 10.7	22	67.0 ± 10.5	41.0
140–159	146.6 ± 4.9	41	63.3 ± 11.3	68.3
120–139	127.8 ± 6.2	51	66.4 ± 10.9	60.8
<120	104.7 ± 8.8	53	65.2 ± 12.1	64.1
Current DBP (mm Hg)	Mean DBP in group (mm Hg)	Number of Patients	Age	Male (%)
All	81.5 ± 13.8	167	65.3 ± 11.3	61.1
≥100	108.3 ± 6.6	18	61.7 ± 12.2	55.5
90–100	92.3 ± 2.8	29	65.1 ± 10.4	58.6
80–89	82.5 ± 2.5	52	65.3 ± 10.4	63.5
<80	69.0 ± 6.9	68	66.5 ± 12.2	61.9

Systolic blood pressure (SBP), diastolic blood pressure (DBP).

**Table 2:** Cohort characteristics.

Characteristic	Number of Patients (N = 167)	% (From N)
Age: median 66 years [59.5; 73]		
Ischemic heart disease	75	44.9
Arterial hypertension	144	86.3
Heart failure	68	40.7
LV EF < 55%	36	21.6
LV EF < 40%	14	8.4
LV DD in grades 2 and 3	31	18.6
Diabetes	72	43.1
Smokers	39	23.4
Vessel wall stiffness	35	21.0
Using statins	99	59.3
Using antihypertension drugs	139	83.2
Using diuretics	71	42.5

LV = left ventricular, EF = ejection fraction, DD = diastolic dysfunction.

**Table 3:** First phase of validation process.

Number of Subjects: 45 (20 Subjects for Phase 1; 25 Subjects for Phase 2)					
Blood Pressure Ranges:					
Blood Pressure Classification	Systolic Blood Pressure (mmHg)		Diastolic Blood Pressure (mmHg)	Subjects in Phase 1	Subjects in Phase 2
Normal	<120	and	<80	5	≥6
Prehypertension	120–139	or	80–89	5	≥6
Stage 1 hypertension	140–160	or	90–100	5	≥6
Stage 2 hypertension	≥160	or	≥100	5	≥6
Gender:					
At least 22 males and 22 females					
Age:					
All subjects must be 18 to 65 years old.					

**Table 4:** Device accuracy report.

Group	Valid N	MAD (mmHg)	MAPD (%)	MD (mmHg)	CP MAD ≤ 5 mmHg (%)	CP MAD ≤ 10 mmHg (%)	CP MAD ≤ 15 mmHg (%)
Total group SBP	167	3.44 [3.05, 3.82]	2.68 [2.4, 2.96]	−0.05 [−0.7, 0.6]	85.6%	98.2%	99.4%
Total group DBP	167	3.21 [2.78; 3.64]	3.97 [3.43; 4.51]	−1.02 [−1.65; −0.38]	83.8%	98.2%	98.8%
Normal SBP	47	3.32 [2.86; 3.77]	3.24 [2.79; 3.69]	−0.3 [−1.38; 0.78]	91.5%	-	-
Normal DBP	47	3.06 [2.28; 3.85]	4.66 [3.46; 5.85]	1.02 [−0.14; 2.18]	87.2%	97.9%	100%
Prehypertension SBP	51	2.94 [2.22; 3.66]	2.34 [1.78; 2.91]	1.29 [0.25; 2.33]	86.3%	98.0%	100%
Prehypertension DBP	51	2.78 [2.01; 3.56]	3.55 [2.49; 4.61]	−0.94 [−2.01; 0.13]	88.2%	98.0%	98.0%
Stage 1 SAH	43	3.7 [2.97; 4.42]	2.56 [2.06; 3.05]	−0.35 [−1.71; 1.01]	83.7%	100%	-
Stage 1 DAH	43	3.3 [2.52; 4.09]	3.72 [2.86; 4.58]	−2.74 [−3.72; −1.77]	81.4%	100%	-
Stage 2 SAH	26	4.19 [2.69; 5.69]	2.53 [1.61; 3.46]	−1.73 [−3.9; 0.44]	76.9%	92.3%	96.2%
Stage 2 DAH	26	4.15 [2.71; 5.59]	3.99 [2.72; 5.25]	−2.0 [−4.08; 0.08]	73.1%	96.2%	96.2%

Systolic blood pressure (SBP), diastolic blood pressure (DBP), systolic arterial hypertension (SAH), diastolic arterial hypertension (DAH), cumulative percentage (CP), mean difference (MD), mean absolute difference (MAD), and mean absolute percentage difference (MAPD). MD, MAD, and MAPD are presented with 95% confidence intervals (CIs).

## Data Availability

No new data were created or analyzed in this study. Data sharing is not applicable to this article.

## Appendix A

**Table A1:** Overall group characteristics.

Mark	Valid, n	Min	Mean ± Standard Deviation	2.5%	Median and 25/75%	97.5%	Max
SBP Korotkov	167	88	130.5 ± 22.94	91.15	130.0 [114.0; 145.5]	173.55	195
DBP Korotkov	167	55	81.51 ± 13.74	60	81.0 [72.0; 90.0]	112	121
SBP Quark	167	90	130.45 ± 22.86	93.15	130.0 [114.5; 145.0]	171.7	201
DBP Quark	167	56	80.5 ± 12.63	60	80.0 [72.0; 85.5]	109.85	120
MD SBP	167	−16	−0.05 ± 4.25	−7.85	0.0 [−3.0; 3.0]	7	14
MAD SBP	167	0	3.44 ± 2.5	0	3.0 [2.0; 4.5]	8.85	16
MAPD SBP	167	0	2.68 ± 1.85	0	2.31 [1.38; 3.46]	6.64	10.14
MD DBP	167	−18	−1.02 ± 4.15	−7.85	−1.0 [−3.5; 1.0]	6.85	16
MAD DBP	167	0	3.21 ± 2.82	0	3.0 [1.0; 4.0]	9	18
MAPD DBP	167	0	3.97 ± 3.53	0	3.23 [1.64; 5.49]	11.09	23.53

**Table A2:** Subgroup with normotension (according to IEEE guidelines).

Mark	Valid, n	Min	Mean ± Standard Deviation	2.5%	Median and 25/75%	97.5%	Max
SBP Korotkov	47	88	103.32 ± 8.24	89.3	103.0 [97.5; 109.5]	117.7	119
DBP Korotkov	47	55	66.06 ± 5.76	57.45	64.0 [61.5; 70.5]	76.7	77
SBP Quark	47	90	103.02 ± 7.91	91.15	100.0 [97.5; 110.5]	117.7	121
DBP Quark	47	56	67.09 ± 5.67	59.15	67.0 [63.0; 70.5]	79.4	83
MD SBP	47	−7	−0.3 ± 3.64	−6	−1.0 [−4.0; 3.0]	5	6
MAD SBP	47	1	3.32 ± 1.53	1	3.0 [2.0; 4.5]	6	7
MAPD SBP	47	0.88	3.24 ± 1.51	0.9	3.12 [1.99; 4.26]	6.3	6.67
MD DBP	47	−5	1.02 ± 3.92	−4,85	0.0 [−2.0; 4.0]	7.7	15
MAD DBP	47	0	3.06 ± 2.64	0	3.0 [1.0; 4.0]	7.7	15
MAPD DBP	47	0	4.66 ± 4.03	0	3.9 [1.74; 6.58]	12.81	22.06

**Table A3:** Subgroup with prehypertension (according to IEEE guidelines).

Mark	Valid, n	Min	Mean ± Standard Deviation	2.5%	Median and 25/75%	97.5%	Max
SBP Korotkov	51	112	126.06 ± 6.68	114.5	125.0 [121.0; 131.0]	138	138
DBP Korotkov	51	68	79.75 ± 4.52	68.5	80.0 [78.0; 82.0]	87.5	88
SBP Quark	51	116	127.35 ± 7.1	118	125.0 [122.5; 131.0]	140.75	152
DBP Quark	51	70	78.8 ± 3.97	71.25	79.0 [77.0; 81.0]	85	90
MD SBP	51	−6	1.29 ± 3.66	−4	1.0 [−1.0; 3.0]	7.75	14
MAD SBP	51	0	2.94 ± 2.54	0	2.0 [1.0; 4.0]	7.75	14
MAPD SBP	51	0	2.34 ± 2.0	0	1.67 [0.82; 3.25]	6.68	10.14
MD DBP	51	−9	−0.94 ± 3.78	−6	−1.0 [−3.0; 1.0]	4.75	16
MAD DBP	51	0	2.78 ± 2.72	0	2.0 [1.0; 4.0]	8.25	16
MAPD DBP	51	0	3.55 ± 3.73	0	2.6 [1.27; 4.82]	10.17	23.53

**Table A4:** Subgroup with stage 1 hypertension (according to IEEE guidelines).

Mark	Valid, n	Min	Mean ± Standard Deviation	2.5%	Median and 25/75%	97.5%	Max
SBP Korotkov	43	120	143.84 ± 7.46	123.2	145.0 [140.5; 150.0]	154	155
DBP Korotkov	43	79	87.07 ± 5.6	79.05	88.0 [81.5; 91.0]	98	99
SBP Quark	43	118	143.49 ± 8.89	121.45	142.0 [140.0; 150.0]	157.9	162
DBP Quark	43	77	84.33 ± 5.35	78	84.0 [80.0; 87.0]	97	97
MD SBP	43	−10	−0.35 ± 4.36	−8.9	−1.0 [−3.5; 3.5]	7	8
MAD SBP	43	0	3.7 ± 2.33	0.05	4.0 [2.0; 5.0]	8.95	10
MAPD SBP	43	0	2.56 ± 1.58	0.03	2.65 [1.36; 3.37]	6.37	6.49
MD DBP	43	−10	−2.74 ± 3.12	−8.95	−3.0 [−4.0; 0.0]	2.95	4
MAD DBP	43	0	3.3 ± 2.52	0	3.0 [1.5; 4.0]	8.95	10
MAPD DBP	43	0	3.72 ± 2.75	0	3.45 [1.64; 4.82]	9.93	10.99

**Table A5:** Subgroup with stage 2 hypertension (according to IEEE guidelines).

Mark	Valid, n	Min	Mean ± Standard Deviation	2.5%	Median and 25/75%	97.5%	Max
SBP Korotkov	26	150	166.27 ± 11.44	150	163.0 [161.0; 170.0]	192.5	195
DBP Korotkov	26	90	103.73 ± 8.86	90	102.0 [98.0; 111.0]	119.75	121
SBP Quark	26	144	164.54 ± 13.81	146.5	160.0 [155.75; 168.75]	196.62	201
DBP Quark	26	86	101.73 ± 9.05	86	102.0 [96.25; 108.75]	116.88	120
MD SBP	26	−16	−1.73 ± 5.27	−14.75	−1.5 [−3.75; 2.75]	4.75	6
MAD SBP	26	0	4.19 ± 3.64	0.62	3.0 [2.0; 4.0]	14.75	16
MAPD SBP	26	0	2.53 ± 2.24	0.33	1.9 [1.19; 2.66]	9.09	10
MD DBP	26	−18	−2.0 ± 5.05	−11.12	−2.5 [−4.0; 0.75]	7.38	8
MAD DBP	26	0	4.15 ± 3.49	0.62	3.0 [2.0; 5.75]	11.75	18
MAPD DBP	26	0	3.99 ± 3.07	0.52	3.11 [1.93; 5.46]	11.11	15.13
